# Academic Achievement in Physics-Chemistry: The Predictive Effect of Attitudes and Reasoning Abilities

**DOI:** 10.3389/fpsyg.2017.01064

**Published:** 2017-06-28

**Authors:** Paulo N. Vilia, Adelinda A. Candeias, António S. Neto, Maria Da Glória S. Franco, Madalena Melo

**Affiliations:** ^1^School of Social Sciences and Centre for Studies in History and Philosophy of Science, University of ÉvoraÉvora, Portugal; ^2^School of Social Sciences and Centre for Educational Research and Psychology, University of ÉvoraÉvora, Portugal; ^3^Faculty of Arts and Humanities, University of MadeiraFunchal, Portugal; ^4^School of Social Sciences and Interdisciplinary Center for History, Culture and Societies, University of ÉvoraÉvora, Portugal

**Keywords:** academic achievement, attitude, reasoning skills, physics education, chemistry education, high-school

## Abstract

Science education plays a critical role as political priority due to its fundamental importance in engaging students to pursue technological careers considered essential in modern societies, in order to face scientific development challenges. High-level achievement on science education and positive attitudes toward science constitutes a crucial challenge for formal education. Several studies indicate close relationships between students’ attitudes, cognitive abilities, and academic achievement. The main purpose of this study is to analyze the impact of student’s attitudes toward the school discipline of Physics and Chemistry and their reasoning abilities on academic achievement on that school subject, among Portuguese 9th grade students using the data collected during the Project *Academic Performance and Development: a longitudinal study on the effects of school transitions in Portuguese students* (PTDC/CPE-CED/104884/2008). The participants were 470 students (267 girls – 56.8% and 203 boys – 43.2%), aged 14–16 years old (μ = 14.3 ± 0.58). The attitude data were collected using the Attitude toward Physics-Chemistry Questionnaire (ATPCQ) and, the Reasoning Test Battery (RTB) was used to assess the students reasoning abilities. Achievement was measured using the students’ quarterly (9-week) grades in the physics and chemistry subject. The relationships between the attitude dimensions toward Physics-chemistry and the reasoning dimensions and achievement in each of the three school terms were assessed by multiple regression stepwise analyses and standardized regression coefficients (β), calculated with IBM SPSS Statistics 21 software. Both variables studied proved to be significant predictor variables of school achievement. The models obtained from the use of both variables were always stronger accounting for higher proportions of student’s grade variations. The results show that ATPCQ and RTB had a significantly positive relationship with student’s achievement in Physics-chemistry, indicating that both attitudinal and cognitive variables should be taken into account on science education as well as in educative intervention.

## Introduction

In modern societies, science is increasingly a central aspect of our work and our everyday lives. Educators, policymakers, and researchers are focusing on ensuring that science education continues to help preparing future citizens scientifically literate and engaged prone to engage with science in their lives, allow the societies to meet and overcome the news challenges they are facing (Tytler, 2014).

Students’ academic performance is a fundamental indicator to be taken into account when defining and planning educational intervention both at nationwide level (e.g., curriculum definition) and at classroom level (e.g., teaching strategy). However, although it is well established that academic performance is a complex and multivariate issue with numerous variables contributing simultaneously as predictors for its’ explanation most researchers tend to analyze each variable separately (Ozel et al., 2013), preventing them from getting a full picture of the situation (Byrnes and Miller, 2007).

School and society, in general, tend to assign cognitive abilities the preponderant role when defining school curricula or when explaining and evaluating student’s success or failure, although the importance of the affective domain in education is acknowledged for a long time. (Efklides, 2009; Kahveci, 2015). Newer approaches to the comprehension of learning processes include a broader range of relevant variables at the personal level such as metacognitive knowledge and skills, perceptions of how good is the performance in learning, attitudes, emotions, and motivation (Efklides, 2011). The relational aspects of school living, in particular the importance of positive and supportive teacher-student relationships is another area including relevant variables to understand achievement on students with both typical (Longobardi et al., 2016) and atypical development (Prino et al., 2016) These new set of variables are thought to play a relevant role in students’ process of developing a meaningful understanding of scientific concepts (Nieswandt, 2007).

In this paper, we use a multivariate approach to analyze the contribution of both cognitive (measured through a battery of reasoning tests) and affective abilities (measured through an attitude questionnaire) as predictors of achievement in the subject of Physics-chemistry, among Portuguese 9th grade students.

The concept of “intelligence” can be considered as a complex ability to think, to infer, to understand, to solve new problems, to recognize and build structures, relationships and context meanings (Rindermann, 2007). In educational context this general ability to learn meanings and establish and implement relations in various performance situations assumes a particularly important role (Soares et al., 2015) since there is a broad consensus that academic achievement is statistically correlated with students’ cognitive capabilities (Candeias et al., 2007; Deary et al., 2007), with general intelligence being considered the strongest predictor of scholastic achievement (Roth et al., 2015).

Intelligence tests are widely used by educational psychologists to help in the diagnostic and prognostic of students’ cognitive capabilities and difficulties (Watkins et al., 2007) and also to provide students with self-information helping them in the vocational choices (Lemos et al., 2010). Intelligence test results are positively correlated with school grades and also exhibit good predictive abilities as predictors of school achievement (Deary et al., 2007). Some caution should nonetheless be exerted when analyzing these results once academic achievement cannot be exclusively explained by cognitive abilities or other personal variables (Byrnes and Miller, 2007; Lemos et al., 2008) and also because there are evidence that cognitive abilities are themselves shaped by educational experiences (Watkins et al., 2007; Lemos et al., 2008).

Roth et al. (2015) conducted a psychometric meta-analysis on the correlation between standardized intelligence and school grades including a total of 240 independent samples with over 100,000 participants and found a mean correlation of 0.54 in line with previous reviews (e.g., Sternberg et al., 2001) in their analysis the highest correlations values were attained when the tests used rely on both verbal and non-verbal indicators. Concerning school subjects, the highest correlations were found in the mathematics-science subgroup followed by the language subgroup (Roth et al., 2015).

In Portugal, using the same battery of cognitive tests used in this paper – Reasoning Test Battery (RTB) developed by Almeida and Lemos (2006) and Lemos et al. (2010) refer results similar to Roth’s with highest correlations found for the school subjects of Portuguese Language and Mathematics. The RTB version for the 9th grade consists of five tests allowing the assessment of five reasoning dimensions: numerical, verbal, spatial, abstract, and mechanical.

Despite the importance of the cognitive abilities, other personal variables namely in the affective domain, such as attitudes should also be considered since some studies suggest that attitudinal and motivational factors affect cognitive learning and, in this way, they contribute to improving our ability to explain and predict school achievement, as mentioned earlier.

In this study data from a questionnaire developed to measure the students’ attitudes toward learning Physics-chemistry was used to assess the contribution of attitudinal variables in explaining school achievement in that school subject.

Students’ attitudes toward science have received constant attention in the field of science education for several decades although the precise definition of attitude in this field of study is still a matter of debate, hindering the description and measurement of attitudes (Kind et al., 2007). In this study we will consider “attitudes toward science” in agreement with the definition proposed by Osborne et al. (2003): “feelings, beliefs, and values about an object that can be the enterprise of science, school science, the impact of science on society or scientists themselves” (p. 1053) (emphasis added).

When conducting research on the students’ attitudes toward science two important clarifications should be made. First, it is important to distinguish between attitudes toward science in general and attitudes toward school science subjects or activities since it is the perceptions and feelings about the later that are more likely to influence students’ learning and to be significant in determining their decisions about continuing to study sciences or to pursue future careers in this area (Nieswandt, 2005; Tytler and Osborne, 2012). Second, it is important to distinguish between attitudes toward school science and attitudes toward the various science school subjects, since considering attitudes toward the different science subjects in a unified way may cause biased results because students may have different attitudes toward each of them (Can and Boz, 2012).

In Portugal, compulsory education encompasses basic education and secondary education. Basic education lasts for 9 years and is divided into three cycles: the first corresponds to the first 4 years of schooling; the second comprises the next two and, the third that lasts for 3 years (7th to 9th) and corresponds to Lower Secondary Education. In these cycles, the disciplines and the curriculum are common to all students. Science education in the first and second cycles corresponds to a single, general, integrated subject area. In the third cycle science is taught as two separate subjects: Natural Science covering biology and geology themes and Physics and Chemistry, a single subject encompassing physics and chemistry topics. In the 9th year, to which the data used in this study refer, this subject includes concepts of chemistry like the Periodic Table, the basis of atomic structure or chemical bonding and physics themes such as forces, movement, and electricity.

Although physics and chemistry are the two science subjects toward which students’ attitudes are more negative (Tytler and Osborne, 2012) the number of studies referring specifically to students’ attitudes toward these school disciplines isn’t very large (Kahveci, 2015). The published work on these subjects follows the general trend of school science attitudinal studies. Some examples are: the reduction in students’ attitudes toward studying physical science in post-compulsory school levels (Tytler and Osborne, 2012), the gender gap between boys and girls attitudes toward learning chemistry (Cheung, 2009; Can and Boz, 2012) and toward physics (Atasoy et al., 2014), and the relation attitudes and achievement (Bennett, 2001; Salta and Tzougraki, 2004; Kan and Akbaş, 2006).

The “Attitude toward Physic-chemistry questionnaire” (ATPCQ) used in this study was developed by Neto et al. (2011) and assess four attitudinal dimensions: a positive emotions factor referring to the pleasant sensations aroused by studying or attending Physic-chemistry classes, a negative emotions factor relative to the disagreeable feelings induce by this school discipline, a competence factor associated with the capacity of being skilful or successful on this subject and the related activities and, finally utility factor concerning the perceived utility of Physics-chemistry for daily life (e.g., Neto et al., 2011).

The purpose of this study is to analyze the contribution of the five types of reasoning measured by the RTB, and the four attitudinal dimensions assessed by the Attitude toward Physic-chemistry questionnaire, on the 9th grade Portuguese students’ achievement in the Physics-chemistry, using correlational analysis and a multivariate approach based on multiple linear regressions.

## Materials and Methods

### Participants

The data for this study were collected as a part of a larger research project – “Academic Performance and Development: a longitudinal study on the effects of school transitions in Portuguese student” (PTDC/CPE-CED/104884/2008), aimed at assessing the effects of numerous of variables on the achievement of Portuguese basic education students.

This study was conducted using the data collected on the 9th grade students, attending the discipline of Physics-chemistry from 10 Portuguese schools. Schools were selected to represent all three administrative, educational regions: North (3 schools), Center (3 schools), and South (3 schools) in mainland Portugal and the Azores (1 school). The sample consisted of 470 students (267 girls – 56.8% and 203 boys – 43.2%), representing approximately 0.5% of all 9th grade Portuguese students. **Table 1** shows the students’ gender and age distribution by school zone.

**Table 1:** Student gender and age distribution by school zone.

	Gender						Age		
	Female		Male		Total				
School zone	Count	%	Count	%	Count	%	Mean	Std. Dev.	Median
North (3 schools)	97	36.3	73	36.0	170	36.2	14.18	0.44	14.0
Center (3 schools)	65	24.3	39	19.2	104	22.1	14.31	0.54	14.0
South (3 schools)	68	25.5	58	28.6	126	26.8	14.37	0.60	14.0
Azores (1 schools)	37	13.9	33	16.3	70	14.9	14.66	0.76	14.0
Total	267		203		470		14.33	0.58	14.0

### Instruments

#### Reasoning Test Battery (RTB)

The RTB (Almeida and Lemos, 2006) is a set of tests aimed at assessing cognitive achievement considering both inductive reasoning, which is the apprehension of relations between elements, and deductive reasoning, i.e., the application of the inferred relations to new situations. Being originally based on the “*Tests de Raisonnement Différentiel*” (Meuris, 1969) it was developed and published in Portugal and Brazil (Almeida and Primi, 1996), and it’s validated and assessed for Portuguese 5th to 12th school year students.

The version for the 3rd cycle (7th to 9th grades) of basic education includes five tests:

– Numerical reasoning test (NR), consists of 20 numerical linear or alternating sequences (test duration – 10 min);
– Verbal reasoning test (VR) formed by 25 analogies taking into account relationships between words (test duration – 4 min);
– Spatial reasoning test (SR), composed of 20 series of linear or alternating cubes in motion (test duration – 9 min);
– Abstract reasoning test (AR), consists of 25 analogies involving figures without any apparent meaning (test duration – 5 min);
– Mechanical reasoning test (MR), presents 25 problems associated with everyday experiences, also covering basic knowledge of physics and mechanics (test duration – 8 min).

The tests were applied once in begging of the school year.

The results from different schools were statistically standardized (t-score standardization).

#### Attitude toward Physics and Chemistry Questionnaire

The “ATPCQ” used in this study was developed by Neto et al. (2011) considering that the construct “Attitude toward the Physics-chemistry school subject” has a threefold structure based on the classical three components of attitudes: the cognitive, the affective, and the behavioral (Eagly and Chaiken, 1993). However, subsequent factorial analysis of the version for the 3rd cycle of basic education revealed a four-factor structure (e.g., Neto et al., 2011):

– Positive emotions (6 items) includes items related to the agreeable attitudes evoked by studying or attending Physic-chemistry classes.
– Negative emotions (6 items) contains items referring to adverse attitudes induced by this school subject.
– Competence (6 items) is made up of items associated attitudes on the ability to have good results or being skillful when solving problems or performing Physics-chemistry activities.
– Utility (4 items) refers to attitudes about the perceived utility of Physics-chemistry for daily life.

The Attitude toward Physics and Chemistry Questionnaire (ATPCQ) is composed of 22 items, with an answer scale of 4 point Likert type (1 = Strongly Disagree, 2 = Disagree, 3 = Agree, 4 = Strongly Agree).

The questionnaires were applied once in begging of the school year in a single 15-min session.

The results from different schools were statistically standardized (t-score standardization).

#### Academic Achievement

Academic achievement was assessed directly from the students’ school grades at the end of each school term. These data, ranging from 1 to 5, were provided by the schools.

The school grades were chosen to assess achievement due to the lack of national tests on this subject and also because these classifications can be directly related to the students’ academic success (Lemos et al., 2008). According to Roth et al. (2015) school grades are a good measure of school achievement since they include information on scholastic performance over a wide period of time, and based on different sources such as participation in classes or written examinations. In this way they are less prone to error than specific school achievement tests, more subject to temporary mental states and the individual abilities of the examinees (e.g., written versus verbal performance).

### Procedure/Ethics Approval

The surveys, both the RTB and the ATPCQ, were applied collectively in the classroom context, during class time in the presence of the researchers and the class teachers. Each student received a set of documents with a code number including a biographic form, a written informed consent form and the answer sheets for RTB and ATPCQ. Both questionnaires were applied in a single 60–75 min session beginning with RTB.

The participation was volunteer and anonymous. Written informed consent from the parents, authorization from the schools’ directors and authorization from National Committee for the Protection of Data and from Committee for Monitoring Surveys in Schools from the Ministry of Education were obtained before the data collection. All data are confidential and anonymous.

### Data Normalization

The raw data collected for all variables was submitted to a linear normalization procedure and transformed into t-scores, to allow easier comparison and eliminate some ambiguity resulting from differences between the various schools. T-score normalized scores express individual values distance to average in terms of the standard deviation of the distribution. The numerical relations among the normalized scores are exactly same as the raw values, and all the features of the original distribution remain in the distribution of normalized scores (Cohen et al., 2003). The conversion is calculated trough the formula T = 50 + 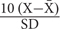, where X is the raw value, 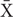 is the mean value and SD is the standard deviation. T-score normalization was originally proposed by W.A. McCall (1922 in Anastasi et al., 2000) and converts the raw data distribution into one with average = 50 and standard deviation = 10. To ensure the correctness of the procedure the mean and standard deviation of each variable were assessed after normalization.

### Data Analysis

Using the normalized data, the existence of statistically significant correlations between the five RTB tests, the four dimensions of ATPCQ and the school grades in the 1st school term was assessed by determining the Pearson Correlation Coefficient (r). The strength of the correlations was classified according to the criteria proposed by Cohen (1988) in which a Pearson correlation value of 0.10–0.29 is small, 0.30–0.49 is medium, and 0.50–1.00 is high. Stepwise multiple linear regressions were then performed to establish a multivariate model of the predictive power of the cognitive and attitudinal data collected on the Physics-chemistry school grades. Standardized versions of the regressions’ B coefficients (β-values) were determined since they provide a measure of the unique explanatory power of the independent variables relative to one another.

This analytical procedure was repeated with the 2nd and 3rd term school grades to determine the stability of both the correlations and the predictive model throughout the year.

For all the multiple linear regressions, independence of residuals was verified by a Durbin–Watson statistic between 1 and 2. Homoscedasticity was verified by visual inspection of plots of studentized residuals versus unstandardized predicted values for each school term. The absence of multicollinearity, as assessed by tolerance values greater than 0.2. The existence of occurring outliers, high leverage points or highly influential points were tested and the occurring studentized deleted residuals greater than ±3 standard deviations, leverage values greater than 0.2, and values for Cook’s distance above 1, were eliminated and the regressions were re-calculated. The assumption of normality was as assessed by inspection of Q-Q plots for each school term.

All the statistical procedures and tests were conducted using the IBM SPSS Statistics 22 software package.

## Results

The correlation analysis between the five RTB tests and Physics-chemistry grades in the 1st school term showed that all the RTB test results were positively and statistically correlated with achievement in Physics-chemistry (**Table 2**). Verbal and numerical reasoning presented the higher correlations (*r* = 0.36 and *r* = 0.33, respectively), though moderate according with Cohen (1988) classification, followed by spatial and abstract reasoning (*r* = 0.28 and *r* = 0.25, respectively) and mechanical reasoning (*r* = 0.17), weak according to Cohen (1988) classification.

**Table 2:** Correlation coefficients between Reasoning Test Battery (RTB) tests and Physics and Chemistry grades.

		RTB NR	RTB VR	RTB SR	RTB AR	RTB MR
1st term	Pearson correlation	0.333^∗∗^	0.359^∗∗^	0.285^∗∗^	0.251^∗∗^	0.173^∗∗^
	Sig. (2-tailed)	0.000	0.000	0.000	0.000	0.000
	*N*	466	469	469	469	469
2nd term	Pearson correlation	0.368^∗∗^	0.368^∗∗^	0.301^∗∗^	0.303^∗∗^	0.206^∗∗^
	Sig. (2-tailed)	0.000	0.000	0.000	0.000	0.000
	*N*	467	470	470	470	470
3rd term	Pearson correlation	0.307^∗∗^	0.350^∗∗^	0.276^∗∗^	0.241^∗∗^	0.173^∗∗^
	Sig. (2-tailed)	0.000	0.000	0.000	0.000	0.001
	*N*	451	454	454	454	454

*^∗∗^Correlation is significant at the 0.01 level (2-tailed).*

The correlation coefficients between the ATPCQ dimensions and the Physics-chemistry grades in the 1st school term were positive and statistically significant for competence, utility and positive emotions and statistically significant but negative for negative emotions. According with Cohen (1988) classification, the correlations with school grades were moderate for both competence and negative emotions (*r* = 0.48 and *r* = -0.32, respectively) and weak for utility (*r* = 0.18) and positive emotions (*r* = 0.11) (**Table 3**).

**Table 3:** Correlation coefficients between ATPQC dimensions and Physics-chemistry grades.

		Positive	Negative		
		emotions	emotions	Competence	Utility
1st term	Pearson correlation	0.106^∗^	-0.323^∗∗^	0.476^∗∗^	0.176^∗∗^
	Sig. (2-tailed)	0.030	0.000	0.000	0.000
	*N*	420	420	420	420
2nd term	Pearson correlation	0.085	-0.350^∗∗^	0.466^∗^	0.142^∗∗^
	Sig. (2-tailed)	0.082	0.000	0.000	0.003
	*N*	421	421	421	421
3rd term	Pearson correlation	0.137^∗^	-0.353^∗∗^	0.453^∗∗^	0.180^∗∗^
	Sig. (2-tailed)	0.006	0.000	0.000	0.000
	*N*	407	407	407	407

*^∗^Correlation is significant at the 0.05 level (2-tailed). ^∗∗^Correlation is significant at the 0.01 level (2-tailed).*

The data assumptions for conducting multiple linear regression were tested as described in the “Materials and Methods” section. Independence of residuals was verified by a Durbin–Watson statistic of 1.88. Data showed homoscedasticity and there were no evidences of multicollinearity. The occurring outliers, high leverage points or highly influential points were eliminated and the regression was re-calculated. The residuals’ normal distribution was verified by inspection of Q-Q plot.

The multiple linear regression model obtained for the 1st term explained 46% of the variance in the Physic-chemistry grades. The competence (β = 0.38) and negative emotions (β = -0.28) dimensions of the attitudes toward Physics-chemistry and verbal reasoning (β = 0.24) were the three largest single significant predictors, with β-value for the negative emotions being negative as expected. The numeric components of reasoning and the utility dimension of attitudes were the other two other variables occurring as significant predictors although with β-values lower than 0.18 (**Table 4**).

**Table 4:** Summary of stepwise multiple regression analysis between RTB and ATPC and Physics and Chemistry 1st term grades.

	*B*	Std. Error	β	*t*	Sig.
Intercept	17.222	3.759		4.582	0.000
Competence	0.374	0.038	0.377	9.888	0.000
Negative emotions	0.245	0.040	0.239	6.184	0.000
RTB – Verbal	-0.275	0.036	-0.275	-7.535	0.000
RTB – Numeric	0.171	0.039	0.174	4.382	0.000
Utility	0.144	0.036	0.145	4.008	0.000

*r^2^ = 0.481; Adjst. *r*^2^ = 0.461; *F*(5,410) = 71.64; *p* < 0.001.*

Considering now the analysis conducted for the 2nd and 3rd school terms, the correlations between each of the five RTB tests and Physics-chemistry grades in both school terms were always positive and statistically significant, with similar values to those found in the 1st term. The order of relevance was also always the same with verbal reasoning presenting the higher correlation values followed by numerical, spatial, abstract, and mechanical reasoning. Although varying very little, all the RTB tests, exhibited the same pattern of variation increasing from the 1st to the 2nd terms and decreasing again in the 3rd (**Table 2**).

Correlation coefficients between ATPCQ dimensions and Physics-chemistry grades in the 2nd and 3rd school terms are shown in **Table 3**. The correlation values were always positive and statistically significant for competence and utility, and statistically significant but negative for negative emotions. For the positive emotions dimension, correlations were always positive but were only statistically significant in the 3rd term. The *r*-values and the relative order of importance of the four ATPCQ dimensions correlations with school grades in the 2nd and 3rd terms were similar to the ones observed in the 1st term and, in the same way as RTB tests correlations, the variation between the three school terms were very small for each of the four ATPCQ dimensions (**Table 3**).

The data assumptions for performing the multiple linear regressions for the 2nd and 3rd terms were tested in the same way as for the 1st semester with the Durbin–Watson statistic being 1.81 and 1.13, respectively. Data showed homoscedasticity, there were no evidences of multicollinearity and the occurring outliers, high leverage points or highly influential points were eliminated and the regressions were re-calculated. The residuals’ normal distribution was verified by inspection of Q-Q plot.

The amount of explained variance was 49 and 41%, respectively, in the 2nd and 3rd terms. The five most relevant statistically significant predictors occurring in both terms were the same as in the 1st term with β-values also very similar to those found for the 1st term (**Tables 5, 6**). In this way, the competence dimension of attitudes continued to be the most important predictor (β = 0.34 in both terms) followed by the negative emotions dimension of attitudes (β = -0.27 in both terms) and by verbal reasoning (β = 0.23 and 0.21 in 2nd and 3rd term, respectively). Numerical reasoning and utility were the two other significant predictors in both terms with β-values lower than 0.18. The abstract component of reasoning and the positive emotions dimension of attitudes occurred as statistically significant predictors in the 2nd and 3rd terms, respectively, but in both cases with a very low β-value of 0.092.

**Table 5:** Summary of stepwise multiple regression analysis between RTB and ATPC and Physics and Chemistry 2nd term grades.

	*B*	Std. Error	β	*t*	Sig.
Intercept	16.222	4.073		3.983	0.000
Competence	0.346	0.038	0.344	9.013	0.000
Negative emotions	-0.272	0.038	-0.269	-7.230	0.000
RTB – Verbal	0.236	0.040	0.227	5.819	0.000
RTB – Numeric	0.180	0.041	0.180	4.387	0.000
Utility	0.092	0.037	0.091	2.510	0.012
RTB – Abstract	0.095	0.042	0.092	2.300	0.022

*r^2^ = 0.500; Adjst. *r*^2^ = 0.492; *F*(6,407) = 67.73; *p* < 0.001.*

**Table 6:** Summary of stepwise multiple regression analysis between RTB and ATPC and Physics and Chemistry 3rd term grades.

	*B*	Std. Error	β	*t*	Sig.
Intercept	16.779	4.515		3.716	0.000
Competence	0.347	0.041	0.342	8.483	0.000
Negative emotions	-0.279	0.040	-0.271	-6.990	0.000
RTB – Verbal	0.221	0.043	0.214	5.171	0.000
Utility	0.139	0.040	0.135	3.507	0.001
RTB – Numeric	0.143	0.042	0.142	3.374	0.001
Positive emotions	0.093	0.039	0.092	2.406	0.017

*r^2^ = 0.419; Adjst. *r*^2^ = 0.410; *F*(6,397) = 47.62; *p* < 0.001.*

As a summary, our results show that the correlation pattern between both the cognitive and attitudinal variables studied and the Physic-chemistry school grades was very similar in all three school terms, with very small changes in the correlation values of all the variables and with the same order of importance occurring in each of the three school terms. The same happened with the predictive models obtained from the multiple linear regressions, where similar amounts of explained variance and the same five principal predictors emerged for the three school terms.

The presence of the positive emotions and the abstract reasoning as significant predictors only in one school term and with relatively weak values, and the fact that two components reasoning (spatial and mechanical) never occurred as significant predictors, were unexpected and deserve a special reference.

## Discussion and Conclusion

The purpose of this study was assessing the contribution of reasoning abilities and attitudes toward the Physics-chemistry discipline on predicting student’s achievement using a multivariate approach, and the analysis of our results indicates that both affective and cognitive variables are relevant predictors of school performance in that school subject. In fact, both the results of the correlation analysis, with statistically significant values occurring for all variables, and the results of the multiple regressions, with the presence of affective and cognitive variables as predictors in the models for all the three school terms, confirm the initial assertion that that school achievement is best predicted when using a multivariate approach including both cognitive and affective variables.

These results are in agreement with others [e.g., Byrnes and Miller (2007) and Lawrenz et al. (2009)] published on this subject both for school science in general and for specific school subjects in particular. Byrnes and Miller (2007) analyzed science achievement in a large sample of 10th to 12th grade North American students using a new framework that stresses the importance of examining a large number of factors in the same study. The variables present in the predictive model include socio-economic status, previous science achievement or “feeling efficacious about graduating high school.” Lawrenz et al. (2009) investigated the variables affecting the physics achievement of 3000 U.S. students, half of which from the 9th grade. The study included different student and teacher/classroom level variables and the final models included, at the student level, variables such as previous knowledge, gender, ethnicity and students’ attitude.

A relevant outcome of our study is the stability of the results throughout the three school terms since it reinforces the confidence in the model obtained as hypothesized initially. In fact, the correlation values found for each reasoning ability and each attitudinal dimension, are similar in all school terms, both concerning the absolute value and the relative order of importance. A parallel situation occurred with the results of the multiple linear regressions, where the significant predictors and their relative order of importance were, in general, the same in each of the three school terms.

Although the study design, with only one data collection for the reasoning abilities and the student’s attitudes, doesn’t allow drawing conclusions about the variation throughout the academic year, the constancy in the results, can also be indicative of the immutability in the student’s school grades over the course of the school year. This may be a matter of concern since it might indicate the school’s incapacity to improve the student’s results and should, therefore, be addressed in future studies.

Considering now in more detail the results of the different analysis conducted, our results show that correlations between the various reasoning abilities assessed by the five RTB tests and Physics-chemistry grades were always statistically significant with values ranging 0.37–0.17. This range of values is somewhat lower than others reported in the literature where 0.5 is considered the average value (Deary et al., 2007; Lemos et al., 2010; Roth et al., 2015), but are comparable to the results reported for 9th grade Portuguese students by Soares et al. (2015) in a study using the same battery of reasoning tests and the science school grades, where the correlations varied between 0.22 and 0.46.

Regarding each of the different reasoning tests, verbal and numeric reasoning always showed the higher correlations with school grades, which is in line with other studies conducted in Portugal using the same tests (Lemos et al., 2010; Soares et al., 2015). The nature of the abilities assessed by these two tests – language knowledge and basic mathematical skill, and their close relation with school learnings, is suggested by the authors as a possible reason for the relative higher importance of these two tests. Roth et al. (2015) found a similar result in their meta-analysis with the verbal reasoning tests presenting the higher correlations with school grades, and offer a closely related explanation when they stress the importance of verbal abilities for the successful participation in class activities and in written examinations, which in turn are fundamental in establishing school grades.

The comparatively lower correlations for spatial, abstract and particularly for mechanical reasoning may be somewhat unexpected, considering the nature of the school subject studied – Physics-chemistry, but are similar to the results found in other studies using this battery of tests (Lemos et al., 2010; Soares et al., 2015). Our results are also in accordance with several others presented by Hegarty (2014) who reviewed the published work on this topic. In her review, Hegarty (2014) refers various studies presenting significant correlations, with values comparable to ours, between achievement in physics and chemistry school subjects and spatial reasoning abilities.

The correlations between the four ATPCQ dimensions and the Physics-chemistry grades were also all statistically significant except for the positive emotions dimension in the 2nd term. As stated earlier, the values for each dimension were similar in all the three school terms and were moderate and positive for competence (0.45 < *r* < 0.48), moderate and negative for negative emotions (-0.32 < *r* < -0.35), and positive and weak in the cases of utility (0.14 < *r* < 0.18) and positive emotions (0.09 < *r* < 0.14). These results are in agreement with previous studies, referring the existence of statistically significant correlations between the attitudes toward the school disciplines of physics and chemistry and achievement. Kingir and Aydemir (2012) in a study with 81, 11th grade Turkish students found a correlation of 0.52 between attitude toward chemistry and school grades. Salta and Tzougraki (2004) examining a sample of 567 11th grade Greek students and also report moderate to low correlations (0.24 < *r* < 0.41 for the different subscales) between attitude toward chemistry and achievement. The differences between their subscales and the ones found in our questionnaire hinder direct comparisons, however, their “importance” subscale (*r* = 0.24) may be comparable to our “utility” dimension and both present similar correlation values. Analyzing the correlations between students’ attitudes toward physics measured through various attitudinal scales Awodun et al. (2014), in their study with senior secondary Nigerian students, obtained a significant correlation of 0.48 and Vahedi and Yari (2014) reported a 0.27 correlation for Iranian high-school students. Veloo et al. (2015) found significant correlations of 0.24 and 0.19 for the “Interest toward Physics” and “Attitude toward difficulty in Physics” subscales, respectively, but didn’t obtain a significant correlation for “Attitude toward the importance of Physics” subscale. Chang and Cheng (2008) also reported a moderate correlation between achievement in physics (*r* = 0.33) and chemistry (*r* = 32) and a measure of self-confidence and interest in science for a sample of 11th grade Taiwanese students. Baran and Maskan (2011) in a study with 396 high-school Turkish students on the relationship between science self-concept and achievement on physics found a significant correlation (*r* = 0.169) between the subscale “interest in science” and physics grades.

To conclude the analysis of the correlations between the four ATPCQ dimensions and the Physics-chemistry grades, we would like to draw attention to the fact that the dimensions “positive emotions” and “utility” have very low correlation values, always less than half of those observed for the other two attitudinal dimensions. This results, although not unexpected [e.g., Cheung (2009) for Hong Kong or Can and Boz (2012) for Tukey], are worth mention because they lead us to question the efficacy of school educational intervention both in promoting positive attitudes toward science and in guaranteeing that students understand the usefulness and applicability of what is taught.

In what concerns the stepwise multiple linear regressions performed to assess the predictive power of the cognitive and attitudinal variables studied on the Physics-chemistry achievement our models explain 41–49% of the variance in school grades. The majority of comparable studies found in the bibliographic research report, values of explained variance lower than ours, normally around or below 20%. Acar et al. (2015) using a sample of 8th grade Turkish students and multiple regression methods, obtained a model explaining 19% of the variance on the scores of a conceptual knowledge test with topics of physics. Prior knowledge, scientific reasoning and utility value of science were the significant predictors. Also in Turkey and also using multiple regressions, Kan and Akbaş (2006) found that 10 % of total variance in the achievement score of chemistry could be explained by the attitudes of students’ toward the chemistry course and that an additional 2% variance was explained by including students’ self-efficacy in the model. Lawrenz et al. (2009) using hierarchical linear modeling on a sample of 9th grade North American students, obtained a model explaining 19% of the variance in a physics achievement test. Attitude toward physics, prior knowledge, and mathematics achievement were the most important predictors included in the model. In addition, Awodun et al. (2014) using multiple regression, report a final model that explains 81% of physics achievement variance of senior Nigerian high-school students, with attitude to physics, study habits and interest in physics as the three most relevant predictors.

The results of our study confirm the relevance of using multivariate approaches when assessing students’ achievement and point the way for future research. The small number of variables used might be considered as a limitation of this work but simultaneously reveal the direction for future studies. We intend to expand our analysis with other potentially relevant variables such as previous knowledge or school-related variables like teaching methods or classroom environment. Another limitation of this study is the data collection design. Future research could include two moments of data collection for attitudes and cognitive abilities one in the beginning and other at the end of the academic year, which will allow a more thorough assessment of the effect of school educational intervention in these areas.

## Author Contributions

Conceptualization: PV, AC, and AN; Methodology: PV, AC, and MM; Formal analysis: AN; Investigation: MF and MM; Writing-review and editing: PV and AC; Supervision: AN.

## Conflict of Interest Statement

The authors declare that the research was conducted in the absence of any commercial or financial relationships that could be construed as a potential conflict of interest.

## References

[B1] Acar Ö. Türkmen L. Bilgin A. (2015). Examination of gender differences on cognitive and motivational factors that influence 8th graders’ science achievement in turkey. *Eur. J. Math. Sci. Technol. Educ.* 11 1027–1040. 10.12973/eurasia.2015.1372a

[B2] Almeida L. S. Primi R. (1996). *Bateria de Provas de Raciocinio (BPR 5)*. Braga: Instituto de Educação e Psicologia.

[B3] Almeida L. S. Lemos G. (2006). *Bateria de Provas de Raciocínio: Manual Técnico*. Braga: Centro de Investigação em Psicologia.

[B4] Anastasi A. Urbina S. Veronese M. A. V. (2000). *Testagem psicológica*. Porto Alegre: Artmed Editora.

[B5] Atasoy Ş. Ergin S. Şen A. I. (2014). The effects of peer instruction method on attitudes of 9th grade students towards physics course. *Eur. J. Phys. Chem. Educ.* 6 88–98. 10.12973/ejpce.2014.00072a

[B6] Awodun A. O. Oni S. A. Aladejana A. L. (2014). Students’ variables as predictor of secondary school students’ performance in physics. *Int. J. Sci. Res.* 4 541–545.

[B7] Baran M. Maskan A. K. (2011). A study of relationships between academic self concepts, some selected variables and physics course achievement. *Int. J. Educ.* 3 10.5296/ije.v3i1.586

[B8] Bennett J. (2001). The development and use of an instrument to assess students’ attitude to the study of chemistry. *Int. J. Sci. Educ.* 23 833–845. 10.1080/09500690010006554

[B9] Byrnes J. P. Miller D. C. (2007). The relative importance of predictors of math and science achievement: an opportunity–propensity analysis. *Contemp. Educ. Psychol.* 32 599–629. 10.1016/j.cedpsych.2006.09.002

[B10] Can H. B. Boz Y. (2012). A cross-age study on high school students attitudes toward chemistry. *Int. J. New Trends Educ. Implic.* 3 82–89.

[B11] Candeias A. Rosário A. Almeida L. Guisande M. (2007). Bateria de provas de raciocínio diferencial: suporte à sua utilização em orientação vocacional. *Rev. Port. Pedagog.* 41 143–156.

[B12] Chang C.-Y. Cheng W.-Y. (2008). Science achievement and students’ self-confidence and interest in science: a taiwanese representative sample study. *Int. J. Sci. Educ.* 30 1183–1200. 10.1080/09500690701435384

[B13] Cheung D. (2009). Students’ attitudes toward chemistry lessons: the interaction effect between grade level and gender. *Res. Sci. Educ.* 39 75–91. 10.1007/s11165-007-9075-4

[B14] Cohen J. (1988). *Statistical Power Analysis for the Behavioral Sciences.* Hillsdale, NJ: L. Erlbaum Associates.

[B15] Cohen J. Cohen P. West S. G. Aiken L. S. (2003). *Applied Multiple Regression/correlation Analysis for the Behavioral Sciences.* 3rd Edn Mahwah, NJ: Lawrence Erlbaum Associates.

[B16] Deary I. J. Strand S. Smith P. Fernandes C. (2007). Intelligence and educational achievement. *Intelligence* 35 13–21. 10.1016/j.intell.2006.02.001

[B17] Eagly A. H. Chaiken S. (1993). “Process theories of attitude formation and change: the elaboration likelihood and heuristic-systematic models,” in *The Psychology of Attitudes* eds Eagly A. H. Chaiken S. (Fort Worth, TX: Harcourt Brace Jovanovich College Publishers) 305–349.

[B18] Efklides A. (2009). The role of metacognitive experiences in the learning process. *Psicothema* 21 76–82. 19178860

[B19] Efklides A. (2011). Interactions of metacognition with motivation and affect in self-regulated learning: the masrl model. *Educ. Psychol.* 46 6–25. 10.1080/00461520.2011.538645

[B20] Hegarty M. (2014). Spatial thinking in undergraduate science education. *Spat. Cogn. Comput.* 14 142–167. 10.1080/13875868.2014.889696

[B21] Kahveci A. (2015). Assessing high school students’ attitudes toward chemistry with a shortened semantic differential. *Chem. Educ. Res. Pract.* 16 283–292. 10.1039/C4RP00186A

[B22] Kan A. Akbaş A. (2006). Affective factors that influence chemistry achievement (attitude and self efficacy) and the power of these factors to predict chemistry achievement-I. *J. Turk. Sci. Educ.* 3 76–85.

[B23] Kind P. Jones K. Barmby P. (2007). Developing attitudes towards science measures. *Int. J. Sci. Educ.* 29 871–893. 10.1080/09500690600909091

[B24] Kingir S. Aydemir N. (2012). An investigation of the relationships among 11th grade students’ attitudes toward chemistry, metacognition and chemistry achievement. *Gazi Univ. J. Gazi Educ. Faculty* 32 823–842. 10.17152/gefd.48837

[B25] Lawrenz F. Wood N. B. Kirchhoff A. Kim N. K. Eisenkraft A. (2009). Variables affecting physics achievement. *J. Res. Sci. Teach.* 46 961–976. 10.1002/tea.20292

[B26] Lemos G. Almeida L. S. Guisande M. A. Primi R. (2008). Inteligência e rendimento escolar: análise da sua relação ao longo da escolaridade. *Rev. Port. Educ.* 21 83–99.

[B27] Lemos G. C. Almeida L. S. Guisande M. A. Barca A. Primi R. Martinho G. Fortes I. (2010). Inteligência e rendimento escolar: contingências de um relacionamento menos óbvio no final da adolescência. *Rev. Galego-Port. Psicol. Educ.* 18 163–167.

[B28] Longobardi C. Prino L. E. Marengo D. Settanni M. (2016). Student-teacher relationships as a protective factor for school adjustment during the transition from middle to high school. *Front. Psychol.* 7:1988 10.3389/fpsyg.2016.01988 PMC5179523 28066305

[B29] Meuris G. (1969). *Tests de Raisonnement Différentiel.* Bruxelles: Editest.

[B30] Neto A. Candeias A. Pomar C. Costa P. Oliveira M. Silva S. (2011). “Questionários de atitudes face à língua portuguesa (QAFLP), matemática (QAFM), ciências da natureza (QAFCdN), ciências naturais (QAFCN) e ciências físico-químicas (qafcfq) em alunos portugueses do ensino básico: estudo psicométrico,” in *Proceedings of the XI Congreso Internacional Galego-Portugués de Psicopedagoxía* (Coruña: Universidade da Coruña).

[B31] Nieswandt M. (2005). “Attitudes toward science: a review of the field,” in *Beyond Cartesian Dualism* eds Cobern W. W. Tobin K. Brown-Acquay H. Espinet M. Irzik G. Jegede O. (Dordrecht: Springer) 41–52. 10.1007/1-4020-3808-9_4

[B32] Nieswandt M. (2007). Student affect and conceptual understanding in learning chemistry. *J. Res. Sci. Teach.* 44 908–937. 10.1002/tea.20169

[B33] Osborne J. Simon S. Collins S. (2003). Attitudes towards science: a review of the literature and its implications. *Int. J. Sci. Educ.* 25 1049–1079. 10.1080/0950069032000032199

[B34] Ozel M. Caglak S. Erdogan M. (2013). Are affective factors a good predictor of science achievement? Examining the role of affective factors based on PISA 2006. *Learn. Ind. Diff.* 24 73–82. 10.1016/j.lindif.2012.09.006

[B35] Prino L. E. Pasta T. Gastaldi F. Longobardi C. (2016). The effect of autism spectrum disorders, down syndrome, specific learning disorders and hyperactivity and attention deficits on the student-teacher relationship. *Electron. J. Res. Educ. Psychol.* 14 89–106. 10.14204/ejrep.38.15043

[B36] Rindermann H. (2007). The big g-factor of national cognitive ability. *Eur. J. Pers.* 21 767–787. 10.1002/per.658

[B37] Roth B. Becker N. Romeyke S. Schäfer S. Domnick F. Spinath F. M. (2015). Intelligence and school grades: a meta-analysis. *Intelligence* 53 118–137. 10.1016/j.intell.2015.09.002

[B38] Salta K. Tzougraki C. (2004). Attitudes toward chemistry among 11th grade students in high schools in Greece. *Sci. Educ.* 88 535–547. 10.1002/sce.10134

[B39] Soares D. L. Lemos G. C. Primi R. Almeida L. S. (2015). The relationship between intelligence and academic achievement throughout middle school: the role of students’ prior academic performance. *Learn. Ind. Diff.* 41 73–78. 10.1016/j.lindif.2015.02.005

[B40] Sternberg R. J. Grigorenko E. Bundy D. A. (2001). The predictive value of IQ. *Merrill-Palmer Q.* 47 1–41. 10.1353/mpq.2001.0005

[B41] Tytler R. (2014). “Attitudes, identity, and aspirations toward science,” in *Handbook of Research in Science Education* eds Lederman N. G. Abell S. K. (New York, NY: Routledge) 82–103.

[B42] Tytler R. Osborne J. (2012). “Student attitudes and aspirations towards science,” in *Second International Handbook of Science Education* eds Fraser B. J. Tobin K. McRobbie C. J. (Dordrecht: Springer) 597–625. 10.1007/978-1-4020-9041-7_41

[B43] Vahedi S. Yari M. (2014). Role of cognitive and emotional factors on educational achievement among high school students in physics. *Eur. Online J. Nat. Soc. Sci.* 3 572–579.

[B44] Veloo A. Nor R. Khalid R. (2015). Attitude towards physics and additional mathematics achievement towards physics achievement. *Int. Educ. Stud.* 8 35–43. 10.5539/ies.v8n3p35

[B45] Watkins M. W. Lei P.-W. Canivez G. L. (2007). Psychometric intelligence and achievement: a cross-lagged panel analysis. *Intelligence* 35 59–68. 10.1016/j.intell.2006.04.005 PMC6526451 31162422

